# Specchio-COVID19: a digital cohort study to improve public involvement in epidemiological research: Helene Baysson

**DOI:** 10.1093/eurpub/ckac129.570

**Published:** 2022-10-25

**Authors:** H Baysson, F Pennacchhio, A Bal, N Pullen, J Lamour, C Semaani, ME Zaballa, C Graindorge, I Guessous, S Stringhini

**Affiliations:** 1 Faculté de Médecine, Universite de Genève, Geneve, Switzerland; 2 Unité D'épidémiologie Populationnelle, Hôpitaux Univesitaires de Genève, Geneve, Switzerland

## Abstract

**Background:**

To manage the sanitary crisis and rapidly assess the seroprevalence of anti-SARS-CoV-2 antibodies in the canton of Geneva, we invited previous participants of an annual health survey of the general population to a first serological test. As the pandemic progressed, it become clear that there would be a significant longer impact on health and wellbeing of population. Moreover, there was a need to assess the adherence of the population regarding COVID-19 prevention measures, over time, as well as to provide scientific knowledge about antibodies dynamics and protection from new infections. For all these reasons, a long-term follow-up has been settled via the dedicated digital platform Specchio-COVID19 and on-line questionnaires and repeated serological tests.

**Methods:**

Several measures were designed to maintain high retention and involvement, including regular electronic newsletters with links to a “News” webpage, a “Research” webpage for dissemination of publications and the organization of webinars specifically dedicated to participants. A specific email address and a dedicated hotline were set up so that participants can get in touch with the Specchio-COVID19 team.

**Results:**

Specchio-COVID19 was launched in November, 2020. Up to February 2022, 10'946 individuals (57% women, median age 48) joined the project. Over time, participation rate remains around 65% for each release of questionnaire. 550 participants (5%) definitely dropped out.

**Conclusions:**

Our digital cohort facilitates participants’ involvement, allowing participation from remote locations, organizing webinar, promoting news and scientific information via newsletters and specific webpages and enabling interaction between researchers and participants.

**Key messages:**

• When designing the Specchio-COVID19 digital cohort, the purpose was not only to collect data.

• But to establish a reciprocal exchange of information between researchers and participants, fostering long-term involvement and health empowerment.

